# Effects of Monovacancy and Divacancies on Hydrogen Solubility, Trapping and Diffusion Behaviors in fcc-Pd by First Principles

**DOI:** 10.3390/ma13214876

**Published:** 2020-10-30

**Authors:** Bao-Long Ma, Yi-Yuan Wu, Yan-Hui Guo, Wen Yin, Qin Zhan, Hong-Guang Yang, Sheng Wang, Bao-Tian Wang

**Affiliations:** 1 Department of Nuclear Science and Technology, School of Energy and Power Engineering, Xi’an Jiaotong University, Xi’an 710049, China; mabaolong@stu.xjtu.edu.cn; 2 Engineering Research Center of Nuclear Technology Application, Ministry of Education, East China University of Technology, Nanchang 330013, China; wuyy20@ecut.edu.cn; 3 Spallation Neutron Source Science Center, Institute of High Energy Physics, Chinese Academy of Sciences, Dongguan 523803, China; guoyh20@sina.com (Y.-H.G.); yinwen@ihep.ac.cn (W.Y.); 4 Department of Reactor Engineering Research & Design, China Institute of Atomic Energy, Beijing 102413, China; zhanqin1983@163.com (Q.Z.); yanghg@139.com (H.-G.Y.); 5 Innovation Center of Extreme Optics, Shanxi University, Taiyuan 030006, China

**Keywords:** first-principles, H solution energy, diffusion barrier, hydrogen bubble

## Abstract

The hydrogen blistering phenomenon is one of the key issues for the target station of the accelerator-based neutron source. In the present study, the effect of monovacancies and divacancies defects on the solution, clustering and diffusion behaviors of H impurity in fcc-Pd were studied through first principles calculations. Our calculations prove that vacancies behave as an effective sink for H impurities. We found that, although the H-trap efficiency of the larger vacancy defect was reduced, its H-trap ability strengthened. There is a short-ranged area around the vacancy defects in which H impurities tend to diffuse to vacancy defects, gather and form hydrogen bubbles. Therefore, the characteristic of large vacancy defects formation in materials should be considered when screening anti-blistering materials for neutron-producing targets or when designing radiation resistant composite materials.

## 1. Introduction

The rapid development of accelerator and neutron source technologies have promoted the application of nuclear technology [1,2]. However, the extreme irradiation environment is a great challenge for nuclear materials [3,4,5]. It is well known that the neutron-producing target is one of the key elements of an accelerator-based neutron source, but at the same time, it meets the severe problems of heat transfer and hydrogen blistering [3,5,6,7,8]. Metals (tungsten, titanium and its alloys, alpha-zirconium and alpha-uranium, stainless steels, etc.) form various lattice defects and metastable phases in a proton irradiation environment, which cause performance degradation or the failure of the materials [5,9,10,11]. Protons deposited in the material would form hydrogen bubbles and degrade the material’s mechanical and heat dispersion performance [5,12,13]. Therefore, the blistering phenomenon in a neutron target leads to the target becoming damaged in a short time, limiting the development of accelerator-based neutron sources [6,14].

To improve resistance against the blistering phenomenon for neutron targets, Kumada et al. [14] designed a three-layered neutron target system for an accelerator-based neutron source, in which palladium (Pd) is used as an anti-blistering material and is placed between the Be target and the Cu heat sink. This kind of “sandwich” target structure can significantly increase the service life of the neutron target, among which the anti-blistering material is very important to the blistering-resistance and overall service life of the target structure [15,16]. Therefore, it is important to clarify the performance of Pd in the proton irradiation environment. The behavior of hydrogen in metals has attracted a lot of research interest over recent years [17,18,19,20]. Various mechanisms have been proposed to explain the hydrogen embrittlement phenomenon of metals such as blistering, hydride phase formation, enhanced local plasticity, and grain boundary weakening [21,22,23,24]. Many experiments have proved that H could induce superabundant vacancy formation (SAV) in many metals such as Pd, Cr and Ni [25,26,27]. The formation of SAV could cause a large volume contraction in metals, which would directly lead to performance degradation or failure of the metallic materials. Moreover, it was found that vacancies may play a crucial role in the hydrogen embrittlement of metals [28,29,30]. Lu et al. [28] reported that vacancies can combine with hydrogen impurities and form a vacancy–hydrogen complex in bulk aluminum, which indicates that vacancies may play a crucial role in the hydrogen embrittlement of this kind of prototypical ductile solid. Liu et al. [31] propounded a vacancy trapping mechanism to explain the formation of hydrogen bubbles and this mechanism has been widely applied to other metal systems. Based on this mechanism, much theoretical work has been done to investigate the multi-H trapping behavior of monovacancies in different materials such as Pd, W, Mo, Be, etc. [12,13,32,33]. However, the dissolution behavior of H impurities in metals is not yet fully understood. Most previous studies focused on the interaction between hydrogen and monovacancies. However, many kinds of defects would be produced during the ion irradiation process, including voids, bubbles, impurity-defect cluster complexes, dislocation defect clusters, dislocation lines, dislocation loops, and precipitates [34]. Therefore, it is necessary to study the interaction between H impurities and vacancy defects with different sizes, and the effect of vacancy scales on hydrogen solubility, trapping, and diffusion behaviors.

To clarify the performance of Pd in the proton irradiation environment, so as to design a better neutron-producing target and improve the lifetime of the target system, the interaction of H impurities and vacancy defects in fcc-Pd was studied through theoretical calculations. First, the solution energies of H impurities in a Pd lattice containing a monovacancy or divacancy were calculated. Considering that ion irradiation would produce point defects with different sizes [3], we also studied the formation of hydrogen bubbles in monovacancies and divacancies. Finally, we evaluated the influence of vacancies on the diffusion behavior of H impurities. The calculation methods and conclusions in this paper help to clarify the kinetic behaviors of H impurities in fcc metals and provide guidance for the design of similar anti-blistering composite materials.

## 2. Computational Methodology

Calculations were carried out based on the pseudo-potential plane wave method through the Vienna Ab initio Simulation Package (VASP 5.4) [35]. The exchange-correlation interaction was described by the Perdew–Burke–Ernzerhof (PBE) function of the generalized gradient approximation (GGA) [36]. We adopted a 108 atom fcc-Pd supercell (3 × 3 × 3—unit cells) and a Gamma-centred 4 × 4 × 4 *k*-point mesh in the calculations [37]. To reliably describe the interactions between H impurities and Pd host atoms, the cutoff energy of plane waves was set to 450 eV. The atomic coordinates relaxation was stopped when the forces on each atom were less than 0.001 eV/Å, at which point the cell shape and volume were also relaxed. The climbing image nudged elastic band (CI-NEB) method [38] was used to study the diffusion paths and corresponding energy barrier of H on fcc-Pd. There are 6 NEB images between the fixed endpoints, the spring constant is −5 eV/A^2^ and the force convergent criteria is −0.03 eV/Å. According to our first principles computations, the equilibrium lattice parameters of fcc-Pd are a = b = c = 3.940 Å, are well agreed with the experimental values [39] (a = b = c = 3.908 Å) and previous calculations (a = b = c = 3.854 Å) [32].

The solution energy of H-interstitial complex defects were calculated by

(1) $$E_{S}=E_{Pd,Int,H}-E_{Pd}-\frac{1}{2}E_{H_{2}}$$

and the energy of H-vacancies complex defects were calculated by

(2) $$E_{S}=E_{\left(N-x\right)pd,V,H}-E_{N-xPd,V}-\frac{1}{2}E_{H_{2}},\;\left(x=1,\;2\right)$$

Where *E_pd,int,h_*, *E_pd_* and $E_{H_{2}}$ represent the total energy of one H dissolve in the interstitial of super-cell Pd, the total energy of super-cell Pd and the energy of H_2_ molecule, respectively; $E_{\left(N-x\right)pd,V,H}$ and $E_{\left(N-x\right)Pd,V}$ are the total energy of one H dissolved in super-cell Pd containing vacancies [*x* = 1 (monovacancy) or *x* = 2 (divacancy)] and the total energy of super-cell Pd containing vacancies, respectively.

The trapping energy (*E_trap_*) of H atoms at the vacancy in Pd was calculated by

(3) $$E_{trap}=E_{\left(N-x\right)Pd,V,nH}-E_{\left(N-x\right)Pd,V,\left(n-1\right)H}-E_{\left(N-x\right)Pd,V,H_{OIS}}+E_{NPd},\;\left(x=1,\;2\right)$$

where $E_{\left(N-x\right)Pd,V,nH}$, $E_{\left(N-x\right)Pd,V,\left(n-1\right)H}$, $E_{\left(N-x\right)Pd,V,H_{OIS}}$, and $E_{NPd}$ represent the total energy of super-cell Pd containing vacancies with *n* H atoms, the total energy of super-cell Pd containing vacancies with *n*−1 H atoms, the total energy of super-cell Pd containing vacancies with one octahedral H, and the total energy of super-cell Pd, respectively.

## 3. Results and Discussion

### 3.1. Dissolution Behavior of H in Interstitial Sites, Monovacancies and Divacancies

**Figure 1:**
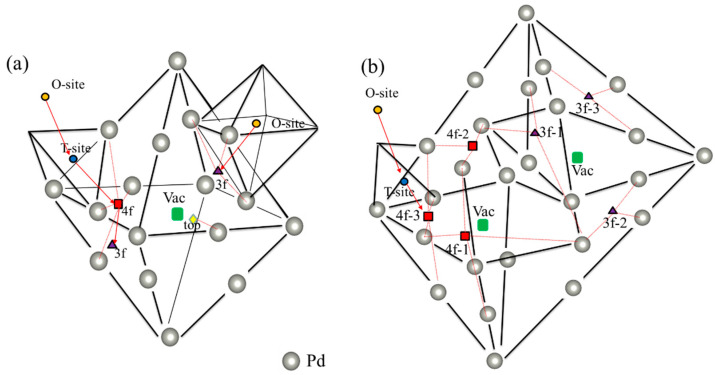
Visualization of relevant interstitial sites at the (**a**) monovacancy and (**b**) divacancy, such as 1vac_top (yellow diamond), 1vac_3f (purple triangle), 1vac_4f (red square), 2Vac_3f−*x* (*x* = 1−3) (purple triangle), 2vac_4f−*x* (*x* = 1–3) (red square), as well as the vacancy-nearing O-site (NOS) (orange circle) and T-site (NTS) (blue circle). The red arrow shows the diffusion path of H atom. The path of (1vac_NOS → 1vac_3f) and (1vac_NOS → 1vac_NOT → 1vac_4f → 1vac_3f) is considered a monovacancy, and the path of (2vac_NOS → 2vac_TOS → 2vac_4f−3) is considered a divacancy.

The solution energy of H impurity at different sites is very important in the thermodynamic analysis of H impurities’ energetic behaviors. In fcc-metal, octahedral (O-site) and tetrahedral (T-site) interstitial sites are two kinds of typical interstitial sites (Figure 1). Table 1 shows that the solution energy of a H impurity in an O-site is lower than that of a T-site, indicating that H is more energetically favorable to occupy the O-site. Besides, the values of solution energies are all negative, indicating that fcc-Pd possesses a certain H-storage capacity, which is consistent with its high hydrogen absorption capacity, as shown by a previous experimental study [40]. The solution energy sfound in the present study is consistent with Nazarov’s calculations [41] and the experimental data of Carstanjen [42].

The vacancy concentration of the Pd sample can reach about 0.02–0.03 at. % [32]. More importantly, various defects (e.g., self-interstitial host atoms and vacancies) are generated during the proton implantation process [43]. Therefore, it is necessary to clarify the solution behavior of H in vacancy defects. According to previous experimental studies [42,44,45], there are different H solution energies in fcc-Pd, which may originate from different solution sites for H impurities at different annealing temperatures. To verify this, two kinds of vacancy size (i.e., monovacancy and divacancy) were considered in this study. The possible sites for H impurities in monovacancies and divacancies are shown in Figure 1a,b, respectively. Table 1 summarizes the corresponding solution energies. As shown in Figure 1, 1vac_site and 2vac_site (site = 3f, 4f and top) represents different high symmetry sites in monovacancies and divacancies, respectively, where 3f, 4f and top sites are along (111), (100) and (110) directions, respectively. It was found that H impurities have different solution energies in different high symmetry sites (Table 1), which is consistent with the dissolution energies measured in previous experiments [42,44]. The lowest solution energies of H in a monovacancy and divacancy were −0.352 eV (1vac_3f site) and −0.416 eV (2vac_4f−3 site), respectively, indicating that H is more energetically likely to stay at 1vac_3f and 2vac_4f−3 sites.

We calculated the solution energies of H impurities in the O-sites near the vacancies to compare the solution preference of H impurities between the interstitial sites (1vac_NOS, the orange circle in Figure 1a) and vacancies (2vac_NOS, the orange circle in Figure 1b). The solution energies of H impurities in both 1vac_NOS (−0.133 eV) and 2vac_NOS (−0.133 eV) were higher than those at 1vac_3f (−0.352 eV) and 2vac_4f−3 (−0.416 eV) sites, indicating that for a H impurity it is more energetically favorable to occupy the vacancies in defected-Pd. Physically, the formation of a vacancy would reduce the surrounding electron density and provide an electron isosurface around the vacancy, where it is more energetically favorable for a H impurity [46,47]. Therefore, H impurities in vacancies always possess a lower solution energy than at other sites.

**Table 1:** Calculated solution energies (*E_S_*) of H in interstitials and vacancies (monovacancy or divacancy). The experimental data are from ref. ^a^ [41], ^b^ [42] and ^c^ [44].

Sites	*E_S_* (DFT) (eV)		*E_S_* (Experiments) (eV)
	This Work (eV)	Others ^a^ (eV)	
O-site	−0.129	−0.11	−0.10 ^b^
T-site	−0.099	−0.09	0.31 ^c^
1vac-3f	−0.352	−0.20	-
1vac-4f	−0.295	−0.16	-
1vac-top	−0.109	0.04	-
1vac-NOS	−0.133	-	-
2vac-3f−1	−0.382	−0.23	-
2vac-3f−2	−0.382	−0.23	-
2vac-3f−3	−0.377	−0.22	-
2vac-4f−1	−0.291	−0.16	-
2vac-4f−2	−0.324	−0.16	-
2vac-4f−3	−0.416	−0.28	-
2vac-NOS	−0.133	-	-

### 3.2. Multiple H Atoms Trapping in Monovacancies and Divacancies

**Figure 2:**
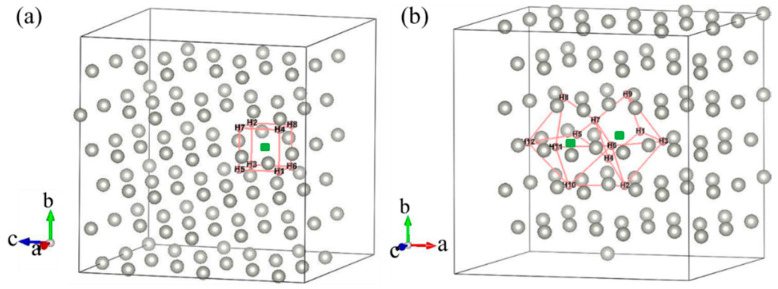
Structure diagram of H dissolved in monovacancy (**a**) and divacancy (**b**) defects of fcc-Pd. The sequence of H bonding positions is shown.

**Figure 3:**
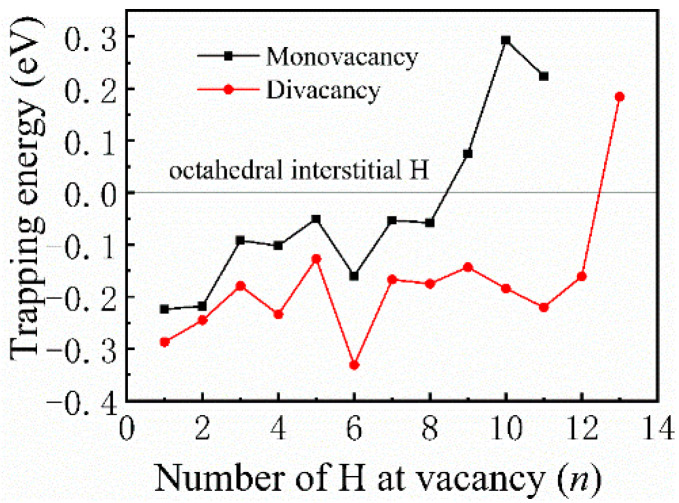
Trapping energies as a function of the number of H atoms embedded in monovacancy and divacancy defects.

Most previous theoretical work focused on H-trap behavior at the monovacancy [13,31,32]. However, more complex defects may be formed during the irradiation process; therefore, H-trap behavior at monovacancies and divacancies were both studied in this study. Figure 2 shows the structure diagrams of different H-vacancy configurations. H atoms were put into the vacancies one by one, and the numbers in Figure 2 represent the sequential order. Figure 3 summarizes the calculation results of trapping energy (*E_trap_*) as a function of H number (*n*). According to the definition of H-trapping energy, the negative trapping energy means a stable *n*H-vacancy complex, that is, H atoms are more inclined to be trapped by the vacancies than dispersed at different O-sites. The trapping energies are negative initially, indicating that H atoms are inclined to be trapped by the vacancies at this stage. As a whole, the trapping energies increase with more implanted H atoms for both monovacancy and divacancy defects, and finally turns from negative to positive, indicating that there is a limit to the number of hydrogen atoms that can be trapped by the vacancies. The number of H atoms that can be trapped in a monovacancy and divacancy are 8 and 12, respectively. More H atoms can be trapped at a divacancy defect, indicating that larger vacancy defects in the lattice could accommodate more H impurities, which may be because the larger vacancy can provide a larger optimal electron density isosurface for H atoms. Notably, a single vacancy can trap up to eight H atoms, and each vacancy in a divacancy defect can trap up to six H atoms on average. That is, although the divacancy defect possesses a stronger H-trapping ability, its H-trapping efficiency is reduced, which may be due to its larger Coulomb repulsion interaction.

### 3.3. The Behavior of H Diffusion in Interstitial Sites, Monovacancies and Divacancies

**Figure 4:**
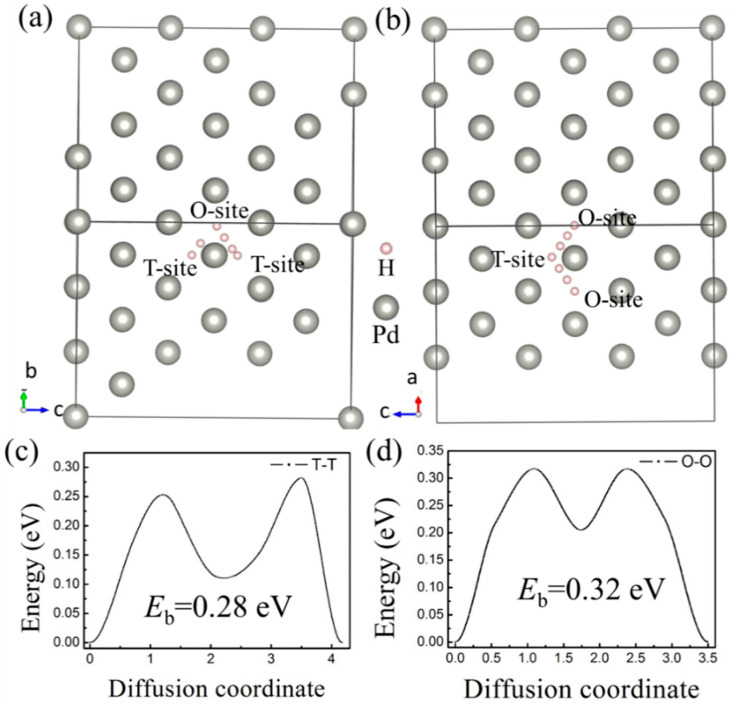
Diffusion pathways from (**a**) T-site to T-site (T-T) and (**b**) O-site to O-site (O-O) in bulk Pd. The corresponding diffusion barrier energy of (**c**) T-T and (**d**) O-O.

H bubbles would form inside or on the surface of a material when the irradiation dose reaches a threshold value, according to experimental studies [3,6,8]. A large-scale diffusion of H impurities inside the lattice is commonly believed to be the origin of hydrogen bubble formation. More importantly, complex defects would be formed under the irradiation environments, which is deemed to be more favorable for the formation of hydrogen bubbles [7]. Therefore, monovacancy and divacancy defects were considered in this paper to study the effect of vacancy defects on the diffusion behavior of H atoms. First, the diffusion barriers of H atoms between the interstitial sites in the ideal Pd-lattice were calculated by the CI-NEB method. H atom diffusion from the T-site to another T-site (expressed as T–T) and from an O-site to another O-site (expressed as O–O) was considered (Figure 4a,b). According to the results, we found that is an intermediate site for both T–T and O–O diffusion pathways. The intermediate site is the O-site for the T–T path, and when the diffusion path is T–T, the H atom would go through an O-site. Therefore, it can be concluded that the diffusion pathways of H impurities among the interstitial sites in fcc-pd are T–O–T and O–T–O, which is consistent with previous results from molecular dynamics (MD) [48] and DFT [49]. The diffusion barriers are 0.28 eV and 0.32 eV for the T–O–T and O–T–O path, respectively.

**Figure 5:**
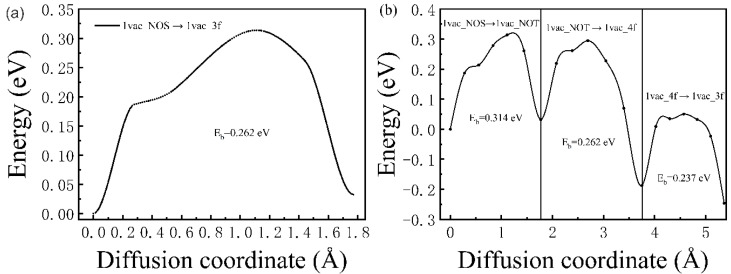
The corresponding diffusion barrier energies of (**a**) 1vac_NOS → 1vac_3f and (**b**) 1vac_NOS → 1vac_NOT → 1vac_4f → 1vac_3f.

The diffusion behavior of H atoms from the interstitial sites to vacancy defects was also studied. For the case of a monovacancy existing in the Pd lattice, two H diffusion paths were considered here, as shown in Figure 1a. Path one is from 1vac_NOS to 1vac_3f (expressed as 1vac_NOS → 1vac_3f); and in path two, the H atom starts from 1vac_NOS, goes through 1vac_NOT and 1vac_4f successively, and reaches 1vac_3f. As shown in Figure 5a,b, the corresponding diffusion barriers ranged from 0.237 to 0.314 eV, which is smaller than the diffusion barriers between interstitial sites in the ideal lattice. Notably, the diffusion barriers of three sub-pathways were 0.314 (1vac_NOS → 1vac_NOT), 0.262 (1vac_NOT → 1vac_4f), and 0.237 eV (1vac_4f → 1vac_3f), respectively (Figure 5b). The closer the atom came to the monovacancy, the smaller the energy barrier was. The energy barrier of 1vac_NOS → 1vac_NOT (0.314 eV) was close to that of the ideal lattice (0.32 eV), which means that the H-trapping ability of vacancies is limited to a short range, and the closer to the vacancy, the stronger the H-trapping ability would have.

**Figure 6:**
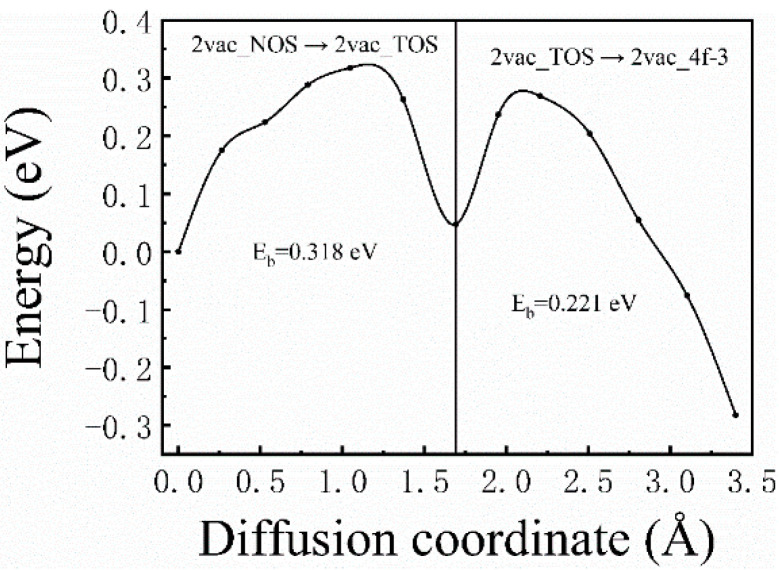
The corresponding diffusion barrier energies of 2vac_NOS → 2vac_TOS → 2vac_4f−3.

For the case of divacancies, one diffusion path was considered, that is, the H atom starting from 2vac_NOS, going through 2vac_NOT, and eventually reaching 2vac_4f−3, which is expressed as 2vac_NOS → 2vac_TOS → 2vac_4f−3 in Figure 1b. The corresponding diffusion barrier energies (Figure 6) were 0.3182 (2vac_NOS → 2vac_TOS) and 0.221 eV (2vac_TOS → 2vac_4f−3), respectively. Similar to the results for the monovacancy defect, the divacancy defect also had a short-range attraction of H atoms, and the energy barrier became smaller when the H atom was closer to the divacancy. However, the diffusion barriers in the case of a divacancy defect are smaller than those in the case of a monovacancy defect, which indicates that the divacancy defect possesses a stronger ability to capture H atoms. Therefore, it can be concluded that the H-trapping ability of the lager vacancy defect is stronger than those smaller vacancy defects. That is, more H atoms would be trapped at large-sized vacancy clusters, which may be the origin of the formation of hydrogen bubbles. Therefore, it is believed that, in addition to the hydrogen diffusion coefficient and thermal conductivity, the characteristic of large vacancy defect formation in materials should be considered when screening anti-blistering materials and designing target systems.

## 4. Conclusions

We performed comprehensive first principles calculations on the effects of monovacancy and divacancy defects on the solubility, clustering, and diffusion behavior of H impurities in fcc-Pd. Our results show that larger vacancy defects possess a stronger H-trapping ability, which is because the larger vacancy defect would significantly reduce the electron density, leading to a smaller H solution energy. Despite large-sized vacancy defects possessing a stronger H-trap ability, its H-trap efficiency would be reduced due to the charge repulsion interaction. Furthermore, we found that the H-trap ability of the vacancy defect is limited to a small surrounding area, as the diffusion barrier of a H impurity significantly reduces inside the effective range and shows a diffusion tendency toward the vacancy. In conclusion, the difficulty of formation of large vacancy defects in materials should be considered when screening anti-blistering materials of neutron source target systems.
